# Down-regulation of frizzled-7 expression decreases survival, invasion and metastatic capabilities of colon cancer cells

**DOI:** 10.1038/sj.bjc.6605307

**Published:** 2009-09-22

**Authors:** K Ueno, S Hazama, S Mitomori, M Nishioka, Y Suehiro, H Hirata, M Oka, K Imai, R Dahiya, Y Hinoda

**Affiliations:** 1 Department of Oncology and Laboratory Medicine, Yamaguchi University Graduate School of Medicine, Ube, Japan; 2 Department of Surgery II, Yamaguchi University Graduate School of Medicine, Ube, Japan; 3 Department of Urology, Veterans Affairs Medical Center and University of California at San Francisco, San Francisco, CA, USA; 4 Sapporo Medical University, Sapporo, Japan

**Keywords:** frizzled-7, siRNA, colorectal cancer, metastasis, RhoA

## Abstract

**Background:**

The canonical Wnt signalling pathway is activated in most sporadic colorectal cancers (CRCs). We previously reported that FZD7 functions as a receptor for the canonical Wnt signalling pathway in colon cancer cells.

**Methods and results:**

In this study, we examined the function of FZD7 in survival, invasion and metastatic capabilities of colon cancer cells. FZD7_siRNA transfection decreased cell viability of HT-29 and HCT-116 colon cancer cells. Expression of c-Jun, phosphorylation of JNK and c-Jun, and activation of RhoA were suppressed after FZD7_siRNA transfection into HCT-116 cells. *In vitro* invasion activity and Wnt target gene expression were also reduced in HCT-116 cells transfected with FZD7_siRNA. Liver metastasis of stable FZD7_siRNA HCT-116 cell transfectants in scid mice was decreased to 40–50% compared to controls. The mRNA levels of FZD7 in 135 primary CRC tissues were examined by real-time PCR. FZD7 mRNA levels were significantly higher in stage II, III or IV tumours than in non-tumour tissues (*P*<0.005), and overall survival was shorter in those patients with higher FZD7 expression (*P*<0.001).

**Conclusion:**

These data suggest that FZD7 may be involved in enhancement of survival, invasion and metastatic capabilities of colon cancer cells through non-canonical Wnt signalling pathways as well as the canonical pathway.

The Wnt signalling pathway is composed of canonical and non-canonical signals. The canonical Wnt signalling pathway that regulates cell fate and proliferation is initiated by binding of Wnt ligands to frizzled transmembrane receptors, and low-density lipoprotein receptor-related proteins. *β*-Catenin associates with T-cell factor (Tcf)/lymphocyte enhancer transcription factors to activate target genes that are related to cell survival, proliferation and invasion (Moon *et al*, 2004; Clevers, 2006). The non-canonical Wnt signalling pathway consists of Wnt/Ca^2+^ pathway and Wnt/c-Jun N-terminal kinase (JNK) (planar cell polarity) pathway (Cohen *et al*, 2008). In the Wnt/Ca^2+^ pathway, Wnt activates intracellular Ca^2+^ signalling, as well as Ca^2+^-dependent protein kinases, such as protein kinase C (PKC) and calmodulin-dependent protein kinase II. In the Wnt/JNK pathway, receptor stimulation activates Dishevelled (Dvl), which in turn activates Rho family of GTPases such as RhoA and Rac. RhoA stimulates c-Jun expression through phosphorylation of c-Jun by Rho-associated kinase (ROCK) (Marinissen *et al*, 2004). Accumulating evidence suggests that non-canonical Wnt signalling is important in regulating cell polarity and movement (Veeman *et al*, 2003).

It is well known that the canonical Wnt signalling pathway is activated in most sporadic colorectal cancers (CRCs; ∼80%), which is mainly caused by mutations in the adenomatous polyposis coli (*APC*) gene (Segditsas and Tomlinson, 2006; Schneikert and Behrens, 2007). In a small proportion of cases (∼10%), activated mutations of the *β*-catenin gene (*CTNNB1*) are found (Schneikert and Behrens, 2007). However, recent findings revealed that Wnt ligands or inhibitors could affect the growth and survival of colon cancer cells in spite of the presence of *APC* or *CTNNB1* mutations (Bafico *et al*, 2004; Suzuki *et al*, 2004; He *et al*, 2005). These findings suggested that Wnt ligands and receptors that function upstream of APC might have a vital role in the development of CRCs.

We have recently reported that frizzled-7 (FZD7), 1 of 10 members of the *FZD* gene family, is predominantly expressed in colon cancer cells and is implicated in canonical Wnt signalling in colon cancer cells with *APC* or *CTNNB1* mutations (Ueno *et al*, 2008). Moreover, the down-regulation of FZD7 with Small-interfering RNA (siRNA) in colon cancer cells resulted in decreased *in vitro* invasion activity (Ueno *et al*, 2008), which is consistent with previous findings that inhibition of FZD7 expression with dominant-negative mutant construct or siRNA reduced the motility of hepatocellular carcinoma cells (Merle *et al*, 2004) or colon cancer cells (Vincan *et al*, 2007), respectively. These data suggest that FZD7 may be important in the invasion and metastasis of CRC. Recent studies have shown that non-canonical Wnt signalling pathways affect the motility and invasion of cancer cells (Weeraratna *et al*, 2002; Croft *et al*, 2004; Qiang *et al*, 2005), but there is little data on CRC cells. Although there is a report that conditional ROCK activation of colon cancer cells induced *in vitro* motility and *in vivo* tumour cell dissemination in nude mice (Croft *et al*, 2004), the relation of FZD7 with non-canonical signals in CRC cells remains unknown.

In this study, we hypothesised that FZD7 may be involved in progression of CRC probably through both canonical and non-canonical signalling pathways. To address this hypothesis, we attempted to reveal a function of FZD7 in the survival, invasion and metastatic capabilities of colon cancer cells with the use of newly prepared and selected siRNAs against FZD7. Moreover, the expression level of FZD7 mRNA was quantitatively evaluated in primary CRC tissues (*n*=135) to clarify whether it could be of prognostic significance for CRC.

## Materials and methods

### Cell cultures

Human colon cancer cell lines, HCT-116 and HT-29, were purchased from the ATCC (Manassas, VA, USA). Human embryonic kidney 293T cells were purchased from RIKEN BRC (Tsukuba, Japan). HCT-116 and HT-29 cells were cultured in McCoy's 5A medium (Gibco/Invitrogen, Carlsbad, CA, USA) supplemented with 10% heat-inactivated fetal bovine serum, 100 IU ml^−1^ penicillin and 100 *μ*g ml^−1^ streptomycin (Sigma, St Louis, MO, USA). 293T cells were cultured in DMEM (Nissui Pharmaceutical, Tokyo, Japan) supplemented with 10% heat-inactivated fetal bovine serum (ATCC), 100 IU ml^−1^ penicillin and 100 *μ*g ml^−1^ streptomycin (Sigma).

### RNA interference

Small-interfering RNAs were constructed in the piGENE hU6 Vector (Clontech, Palo Alto, CA, USA). The nucleotide target sequence for FZD7 were as follows: siRNA1 sequence, gcaccatcatgaaacacgacg; siRNA2 sequence, gcagacgtgcaagagctatgc; siRNA3 sequence, acctcttcataggcacgtcct; siRNA4 sequence, gttctcacctacctggtggac; siRNA5 sequence, ctgcagacgtgcaagagctat; siRNA6 sequence, tacctgatgaccatgatcgtc; siRNA7 sequence, ctctgttcgtctacctcttcatagg; siRNA8 sequence, gtcattctgtctctcacttggttcc; siRNA9 sequence, cctgatgtactttaaggaggaggagag; siRNA10 sequence, gtaaagtgtacaagttacttt; siRNA11 sequence, agcagtggtcaaaccataa; siRNA12 sequence, gaaggttgagaccagcagag; siRNA13 sequence, gggactgtgagcgatccccctgctgc; EGFP siRNA sequence, ggctacgtccaggagcgcacc; scramble siRNA sequence, gatcagcagctgacaacagtatcac. pcDNA3.1-U6EGFP_siRNA and pcDNA3.1-U6FZD7_siRNA8 were obtained by subcloning a *Eco*RI–*Hin*dIII fragment from piGENE-EGFP and piGENE-FZD7_siRNA8 into the *Eco*RI–*Hin*dIII site of pcDNA3.1 (Invitrogen).

### Luciferase reporter assay

For Tcf luciferase assays, cells were transfected with 0.475 *μ*g of TOPflash (Upstate, Lake Placid, NY, USA) and 0.025 *μ*g of pRL-TK Vector (Promega, Madison, WI, USA) according to the manufacture's instructions. The total amount of DNA was adjusted to equal amounts with empty vector. At 48 h after transfection, the luciferase levels were measured by using the Dual-Luciferase Reporter Assay System (Promega). Data are presented as mean values and s.d. for three independent experiments and compared with the level of luciferase activity obtained in the presence of EGFP_siRNA transfectant cells that is represented as 1.

### Crystal violet stain

HT-29 and HCT-116 cells were transfected with plasmid by the Nucleofector system (Amaxa, Cologne, Germany). Cells were seeded in 2 ml medium in a six-well tissue culture plate. At 6 days after transduction, cells were stained with 0.5% crystal violet in 20% methanol for 10 min. Stable transfectants were seeded at 10^4^ cells per well in 2 ml medium in a six-well tissue culture plate. At 8 days after seeding, cells were stained with 0.5% crystal violet in 20% methanol for 10 min. Cell viability was determined by absorbance measurements at 595 nm using 2030 ARVO X4 (PerkinElmer, Boston, MA, USA).

### Cell-cycle assay

HCT-116 cells were transfected with scramble siRNA or FZD7_siRNA8. At 48 h after transfection, cells were harvested, fixed with 75% ethanol for 2 h at 4°C, washed with phosphate-buffered saline (PBS), treated with 100 *μ*g ml^−1^ RNase (Sigma) for 30 min at 37°C and stained in 10 *μ*g ml^−1^ propidium iodide (Sigma) for 30 min at 4°C. Analysis was performed on Cytomics FC500 using FC500 CXP Cytometer software (Beckman Coulter Co., Miami, FL, USA).

### Western blot analysis and RhoA activation assay

At 48 h after transfection, cells were washed in ice-cold PBS and re-suspended in cold buffer containing 1% Nonidet P-40, 0.5% sodium deoxycholate, 0.1% SDS, 0.1 mg ml^−1^ phenylmethylsulfonyl fluoride. Re-suspended cells were passed through the 21-gauge needle to shear the DNA and incubated for 60 min on ice followed by centrifugation at 10 000 ***g*** for 10 min at 4°C. Total protein (10 *μ*g) was analysed by western blotting using primary antibodies and anti-mouse and anti-rabbit IgG HRP-conjugated secondary antibodies (Dako, Glostrup, Denmark), and were visualised with LumiGLO Reagent and Peroxide (Cell Signaling Technology, Beverly, MA, USA). Results of western blot analysis were shown as ratio of band intensity, which indicates the ratio of band intensity of tested protein to that of *β*-actin. Band intensity was measured by using ImageJ software (NIH, Bethesda, MD, USA). At 48 h after transfection, cells were assayed for Rho activation with a Rho Activation Assay Kit (Upstate). The GTP-bound fraction was monitored by western blot analysis. The primary antibodies used were mouse anti-V5 (Invitrogen), anti-*β*-actin (Abcam, Cambridge, UK), anti-p38, anti-phospho-p38, anti-ERK, anti-phospho-ERK, anti-JNK, anti-phospho-JNK, anti-c-Jun and anti-phosphor-c-Jun (BD Biosciences, San Jose, CA, USA) monoclonal antibodies, and rabbit anti-RhoA monoclonal antibody (Cell Signaling Technology).

### Cell invasion assay

Matrigel (1 : 5; BD Biosciences) was added to Transwell membrane filter inserts (8.0 *μ*m pore size; Costar, Cambridge, MA, USA) and incubated for 5 h at 37°C in a 5% CO_2_ tissue culture incubator. HCT-116 cells were transfected with scramble siRNA or FZD7_siRNA8. At 24 h after transfection, cells were harvested and re-suspended in serum-free medium. Aliquots (10^5^ cells) of the prepared cell suspension were added into the upper chamber and the lower chamber was filled with 600 *μ*l of culture media containing 5 *μ*g ml^−1^ fibronectin (Sigma), as an adhesive substrate. Cells were incubated for 48 h at 37°C in a 5% CO_2_ tissue culture incubator. Invasive cells were stained with Diff-Quick solution (Fisher Scientific, Pittsburgh, PA, USA). Cells were counted with a microscope. The average number of cells in five fields per membrane was counted in triplicate.

### Quantitative PCR

Total RNA was isolated from colon cancer cell lines and primary colorectal tumour and non-tumour tissues using the RNeasy Plus Mini Kit (Qiagen, Hilden, Germany) and All prep DNA/RNA Mini kit (Qiagen), respectively. The extracted total RNA was reverse-transcribed into single-stranded cDNA using a High-Capacity cDNA Archive Kit (Applied Biosystems, Warrington, UK). Real-time PCR was performed using first-strand cDNA with TaqMan Universal PCR Master Mix (Applied Biosystems). The assay numbers for the endogenous control (*β*-actin) and target genes were as follows: 4326315E (*β*-actin); Hs00275833_s1 (FZD7). Quantitative PCR was performed on an ABI Prism 7900HT Sequence Detection System (Applied Biosystems). Quantitative PCR parameters for cycling were as follows: 50°C for 2 min hold, 95°C for 10 min 40 cycles of PCR at 95°C for 15 s and 60°C for 1 min. All reactions were carried out in a 20-*μ*l reaction volume in triplicate. For target gene assays, total RNA was isolated from colon cancer cell lines using the RNeasy Plus Mini Kit (Qiagen) 48 h after transfection. The extracted total RNA was reverse-transcribed into single-stranded cDNA using High-Capacity cDNA Archive Kit (Applied Biosystems). Real-time PCR was performed using first-strand cDNA with Power SYBR Green PCR Master Mix (Applied Biosystems). The primers used were as follows: FZD7 forward primer, 5′-ttctcggacgatggctacc-3′; FZD7 reverse primer, 5′-gaaccaagtgagagacagaatgacc-3′; CD44s forward primer, 5′-tcatagaagggcacgtggtg-3′; CD44s reverse primer, 5′-tgggaggtgttggatgtgag-3′; CD44v6 forward primer, 5′-cccagaaggaacagtggtttg-3′; CD44v6 reverse primer, 5′-agctgtccctgttgtcgaatg-3′; CD44v8-9 forward primer, 5′-caggtttggtggaagatttgg-3′; CD44v8-9 reverse primer, 5′-tgtcagagtagaagttgttggatgg-3′; Met forward primer, 5′-caagaggagccccaccttatc-3′; Met reverse primer, 5′-ggcagtattcgggttgtaggag-3′; Survivin forward primer, 5′-cggttgcgctttcctttc-3′; Survivin reverse primer, 5′-tgttcttggctctttctctgtcc-3′; MT1-MMP forward primer, 5′-agattgatgctgctctcttctgg-3′; MT1-MMP reverse primer, 5′-tgccctgagctcttcgttg-3′; Jun forward primer, 5′-ggaaacgaccttctatgacgatg-3′; Jun reverse primer, 5′-agggtcatgctctgtttcagg-3′. Quantitative PCR was performed on an ABI Prism 7900HT Sequence Detection System (Applied Biosystems). Quantitative PCR parameters for cycling were as follows: 95°C for 10 min 40 cycles of PCR at 95°C for 15 s, and 60°C for 1 min. All reactions were carried out in a 20-*μ*l reaction volume in triplicate. The mRNA expression level was determined using the 2^−ΔCT^ method.

### Stable transfectants

pcDNA3.1-U6EGFP_siRNA or pcDNA3.1-U6FZD7_siRNA8 were transfected into HCT-116 cells by FuGENE HD (Roche Diagnostics, Basel, Switzerland). Stably transfected cells were selected 72 h after transfection by neomycin resistance using G418 sulphate (500 *μ*g ml^−1^; Sigma). G418-resistant colonies were cloned by limiting dilution. The expression level of FZD7 mRNA in transfectants was assayed by real-time PCR.

### Liver metastasis in scid mouse

Transfectants were harvested, washed and then re-suspended in serum-free McCoy's 5A medium for injection. Scid mice were injected with Nembutal as an anaesthetic, laid down dorsally and an incision was made about 1 cm long above the spleen using a surgical scissors. A cell suspension (2.5 × 10^6^ per 100 *μ*l) of transfectants was injected into the spleen using a butterfly needle. To arrest the flow of blood when the operator takes the needle out of the spleen, we used an electric knife. The incision was then sutured. Each animal group consisted of seven mice. Three weeks after intrasplenal transplantation, mice were killed and the number of metastatic foci in the liver was counted.

### Primary colorectal cancer tissues and non-tumour tissues

FZD7 mRNA expression was measured in colorectal tumours and non-tumour tissues obtained from 135 patients with CRC after receiving informed consent. The experimental protocol was approved by the institutional ethics committee of the Yamaguchi University Graduate School of Medicine, Ube, Japan. The mean age of patients was 67±11 (mean±s.d.) years, and consisted of both men (70) and women (65). In all patients, the diagnosis of CRC was made on the basis of endoscopic and histological findings. A surgical operation was then carried out. The clinico-pathological characteristics after surgery are shown in Table 1. From the entire surgically resected tissue, viable tumour and non-tumour areas were macroscopically judged and cut out by pathologists. The tissues were immediately frozen in liquid nitrogen or subjected to RNA isolation, which were then stored at −80°C. For RNA storage, RNAlater RNA Stabilization Reagent (Qiagen) was used.

### Statistical analysis

The relationship of FZD7 mRNA levels with clinical stage and follow-up information after surgery was analysed using the Kruskal–Wallis and *post hoc* tests. Kaplan–Meier curves were compared using the log-rank test. Data were processed using GraphPad Prism 5 software (GraphPad Software, San Diego, CA, USA).

## Results

### Preparation and selection of FZD7_siRNA

**Figure 1:**
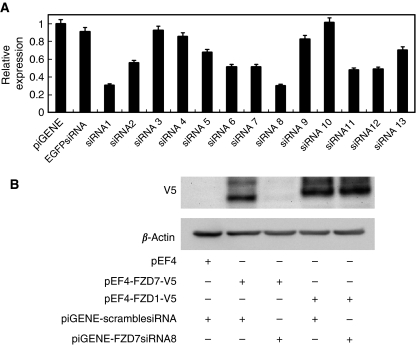
Preparation and selection of FZD7_siRNA. (**A**) Real-time PCR analysis of FZD7 mRNA expression in HCT-116 cells transfected with shRNA expression vectors harbouring siRNA against FZD7. Thirteen siRNAs were designed based on the nucleotide sequence of *FZD7* gene. HCT-116 cells were transiently transfected with shRNA expression vectors. At 48 h after transfection, total RNAs were reverse-transcribed and the levels of mRNA expression of FZD7 were measured by real-time PCR. (**B**) FZD7_siRNA8 specifically inhibited the expression of FZD7. 293T cells were transiently transfected with various combinations of pEF4, pEF4-FZD7-V5, pEF4-FZD1-V5, scramble RNA and FZD7_siRNA8. Cell lysates (10 *μ*g) were analysed by western blotting using anti-V5 or anti-*β*-actin antibodies.

Thirteen shRNA expression vectors harbouring siRNAs against FZD7 were constructed and tested to determine which had the greatest suppressive effect on endogenous FZD7 expression in colon cancer cells. Each shRNA expression vector was transfected into HCT-116 cells and mRNA levels of FZD7 were examined by real-time PCR (Figure 1A). The FZD7 expression in HCT-116 cells was reduced to 30% when using FZD7_siRNA8 compared to a control siRNA. Therefore we used FZD7_siRNA8 as an FZD7_siRNA for the following experiments.

To examine whether the FZD7_siRNA could discriminate between FZD7 and FZD1 with the highest homology, we co-transfected FZD7-V5 or FZD1-V5 and FZD7_siRNA into 293T cells and subjected the whole proteins to immunoblotting with an anti-V5 antibody (Figure 1B). The expression of FZD7-V5 protein was abolished with FZD7_siRNA whereas that of FZD1-V5 was not. Thus the FZD7_siRNA was used to specifically inhibit FZD7.

### FZD7_siRNA suppressed cell viability and invasion

**Figure 2:**
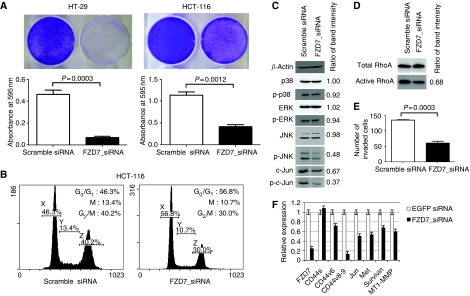
FZD7_siRNA suppressed cell viability and invasion. (**A**) Effect of FZD7_siRNA on cell viability. HCT-116 and HT-29 cells were transiently transfected with FZD7_siRNA or scramble siRNA control. At 6 days after transfection, HCT-116 and HT-29 cells were stained with crystal violet. Cell viability was determined by absorbance at 595 nm. (**B**) Effect of FZD7_siRNA on cell cycle. HCT-116 cells were transfected with FZD7_siRNA or scramble siRNA. Cells were harvested 48 h after transfection and cell-cycle analysis was performed using a Cytomics FC500 cell sorter. (**C**) Effect of FZD7_siRNA on MAP kinase protein expression. HCT-116 cells were transiently transfected with FZD7_siRNA or scramble siRNA. At 48 h after transfection, cytosolic protein (10 *μ*g) was analysed. Expression of p38, phospho-p38, ERK, phosho-ERK, JNK, phospho-JNK, c-Jun or phospho-c-Jun was assessed by western blot analysis. *β*-Actin was used as a loading control. Ratio of band intensity indicates the ratio of band intensity of tested protein to that of *β*-actin. Band intensity was measured by using ImageJ software. (**D**) Effect of FZD7_siRNA on RhoA activation. HCT-116 cells were transfected with FZD7_siRNA or scramble siRNA. At 48 h after transfection, GST-RhoA expression was assessed. Total RhoA was used as a loading control. Ratio of band intensity indicates the ratio of band intensity of tested protein to that of *β*-actin. (**E**) Effect of FZD7_siRNA on cell invasion. HCT-116 cells were transiently transfected with FZD7_siRNA or scramble siRNA. At 24 h after transfection, an aliquot (10^5^ cells) of the prepared cell suspension was added into the upper chamber, the lower chamber was filled with culture media containing fibronectin and cultured for 48 h. Invasive cells were stained and the average number of cells in five fields per membrane was counted in triplicate. (**F**) Effect of FZD7_siRNA on Wnt target gene expression. HCT-116 cells were transiently transfected with FZD7_siRNA or EGFP_siRNA. At 48 h after transfection, total RNAs were reverse-transcribed and the level of mRNA expression of Wnt target genes was measured by real-time PCR.

HCT-116 and HT-29 cells were transfected with scramble siRNA or FZD7_siRNA, and the cells were stained with crystal violet stain 6 days after transfection. Viable cells were decreased to <10 and 40% in HT-29 and HCT-116 cultures, respectively (Figure 2A). On the basis of this result and the fact that we were unable to isolate stable HT-29 siRNA transfectants (see below), we used HCT-116 cells for the following siRNA transfection experiments.

To observe the effect of FZD7_siRNA on cell cycle, we transfected HCT-116 cells with scramble siRNA or FZD7_siRNA, and the cells were analysed by flow cytometry 48 h after transfection. The percentage of cells at G_2_/M phase was reduced from 40 to 30% with FZD7_siRNA transfection (Figure 2B). To evaluate the effect of FZD7_siRNA on the expression and activation of MAP kinases, we subjected HCT-116 cells transfected with scramble siRNA or FZD7_siRNA to immunoblotting. As shown in Figure 2C, the band intensities of c-Jun, p-JNK and p-c-Jun were decreased by FZD7_siRNA.

JNK/c-Jun is involved in the non-canonical Wnt pathway and is regulated by the small GTPase RhoA (Cohen *et al*, 2008). We therefore examined whether FZD7_siRNA affects RhoA activation. HCT-116 cells were transfected with scramble siRNA or FZD7_siRNA and were subjected to the RhoA activation assay. As shown in Figure 2D, FZD7_siRNA decreased RhoA activation. Because RhoA has been shown to be involved in migration and dissemination of colon cancer cells (Croft *et al*, 2004), we assessed, using the Matrigel invasion assay, whether FZD7_siRNA affected the *in vitro* invasion activity of HCT-116 (Figure 2E). The number of invading cells was significantly decreased (*P*<0.0005) with FZD7_siRNA transfection. To assess whether FZD7_siRNA indeed altered Wnt target genes including invasion/metastasis-related ones, we transfected FZD7_siRNA into HCT-116 cells and measured the expression levels of mRNAs with real-time PCR. The transcriptional levels of Wnt target genes including *CD44v8-9* and *MT1-MMP* were decreased with FZD7_siRNA (Figure 2F).

### FZD7_siRNA inhibits *in vivo* metastasis

**Figure 3:**
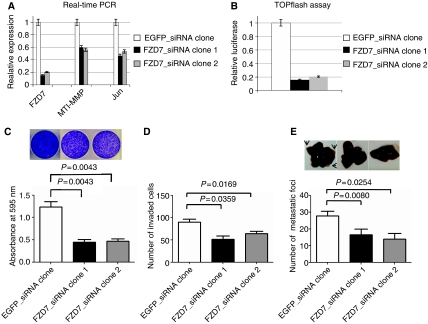
FZD7_siRNA inhibits *in vivo* metastasis. (**A**) The level of mRNA expression of FZD7, MT1-MMP and Jun in stable transfectants expressing FZD7_siRNA. HCT-116 cells were transfected with shRNA-expressing vectors and stable transfectants were selected by neomycin resistance. The expression levels of FZD7, MT1-MMP and Jun mRNAs in transfectants were assayed by real-time PCR. (**B**) Tcf-reporter transcriptional activity in FZD7_siRNA transfectants. Stable transfectants were transiently co-transfected with TOPflash reporter plasmid and pRL-TK plasmid encoding Renilla luciferase as an internal control for transfection efficiency. At 48 h after transfection, cell lysates were measured for relative luciferase activities. Data are presented as mean values ±s.d. for three independent experiments and compared with the level of luciferase activity obtained in the presence of stable transfectants expressing siRNA against EGFP that is represented as 1. (**C**) Cell viability of FZD7_siRNA transfectants. Stable transfectants were seeded at 10^4^ cells per well in 2 ml medium in a six-well tissue culture plate. At 8 days after seeding, cells were stained with crystal violet. Cell viability was determined by absorbance measurements at 595 nm using 2030 ARVO × 4 spectrophotometer. (**D**) Invasive ability of FZD7_siRNA transfectants. Aliquots (10^5^ cells) of the prepared cell suspension were added into the upper chamber and cultured into the lower chamber filled with culture media containing fibronectin for 48 h. Invasive cells were stained and the average number of cells in five fields per membrane was counted in triplicate assay. (**E**) *In vivo* metastatic ability of FZD7_siRNA transfectants. Stable transfectants expressing FZD7_siRNA or EGFP_siRNA were injected into the spleen of scid mice. Three weeks after transplantation mice were killed to count the number of liver metastasis.

HCT-116 cells were transfected with pcDNA3.1-U6EGFP_siRNA or pcDNA3.1-U6 FZD7_siRNA, and the expression level of FZD7 and two target genes (*MT1-MMP* and *Jun*) in stable transfectants was measured with real-time PCR. We used two stable transfectants (FZD7_siRNA clone 1 and clone 2) that show the decreased expression of these genes compared with control cells harbouring EGFP_siRNA for the following experiments (Figure 3A). To test canonical Wnt signal-transducing activity, we transfected TOPflash reporter plasmid into the transfectants, and measured the Tcf transcriptional activity (Figure 3B). We found that Tcf activity was decreased to ∼20% in both FZD7_siRNA clones compared to control cells. Cell viability and invasion activity were also significantly decreased in FZD7_siRNA transfectants (*P*<0.005 and <0.05, respectively; Figures 3C and D).

The anti-metastatic activity of FZD7_siRNA was also shown in an *in vivo* liver metastasis model (Figure 3E). FZD7_siRNA transfectants were transplanted into the spleen of scid mice, and after 3 weeks, the mice were killed to count liver metastasis colonies. Liver metastases in mice transplanted with FZD7_siRNA transfectants were significantly decreased compared to the EGFP_siRNA transfectants (*P*<0.05).

### FZD7mRNA expression levels in primary colorectal tumour tissues

**Figure 4:**
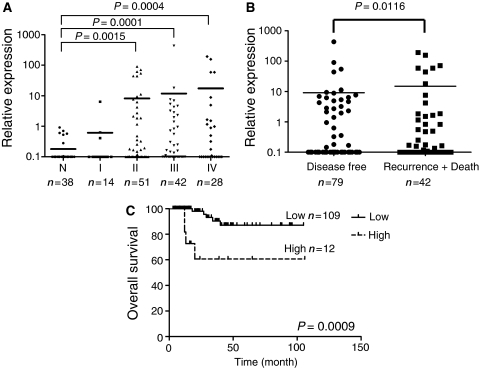
The expression level of FZD7 mRNA in primary colorectal tumour and non-tumour tissues. (**A**) Comparison of tumour with non-tumour tissues. The mRNA levels of FZD7 in primary colorectal tumour and non-tumour tissues were examined by real-time PCR. Tumours were divided into four groups according to the pathological stage (see Table 1). (**B**) Effect of clinical course after surgery on FZD7 expression level. Patients were divided into disease free after surgery and recurrence or death after surgery groups according to the follow-up information after surgery. Disease free indicated a patient group with no recurrence after surgery. Recurrence+Death indicated a patient group with recurrence or death after surgery. (**C**) Kaplan–Meier analysis for overall survival of patients. High or low indicates the patients with the FZD7 mRNA levels ⩾ or < the mean value (11.1) of all tumours tested.

The expression levels of FZD7 mRNA in 135 primary CRC and 38 non-tumour tissues were examined by real-time PCR. The clinico-pathological characteristics of patients are shown in Table 1. FZD7 mRNA expression was significantly higher in the stage II, III or IV tumour tissues than in non-tumour tissues (*P*<0.005; Figure 4A). Follow-up information regarding survival was available for 121 patients. As shown in Table 1, there was no significant difference in clinico-pathological characteristics between this patient group (*n*=121) and total patients (*n*=135). The mRNA level of FZD7 was significantly higher in patients with recurrence or death after surgery than in those with no recurrence (disease free) after surgery (*P*<0.05; Figure 4B). Although there was no association of FZD7 mRNA expression level with age, sex, tumour grade, lymph node metastasis, distant metastasis or histological type on univariate analysis (data not shown), higher FZD7 mRNA expression (⩾mean value of all tumours tested) was significantly associated with shorter survival (*P*<0.001; Figure 4C).

## Discussion

Transient transfection of FZD7_siRNA prepared for this study into colon cancer HCT-116 or HT-29 cells gave rise to a significant suppression of cell viability (Figure 2A), confirming our previous finding (Ueno *et al*, 2008). This also seems to be consistent with a previous finding that siRNA inhibition of FZD7 decreased the viability of mesenchymal stem cells (hMSCs; Song *et al*, 2006). However, in contrast to this report, no apoptotic cells were detected in our present experiments as well as in our previous studies (Ueno *et al*, 2008). Although the mechanism is not fully understood at present, the decrease of G_2_/M cells (Figure 2B) suggests the involvement of the *β*-catenin/Tcf target genes *c-myc* and *cyclin-D* (He *et al*, 1998; Tetsu and McCormick, 1999). Our previous data demonstrated that the expression levels of c-myc and cyclin-D mRNAs increased after FZD7 transfection into HCT-116 cells and a siRNA against FZD7 suppressed c-Myc protein expression (Ueno *et al*, 2008). Conversely, some Wnt signals were shown to promote viability in some cell types (Almeida *et al*, 2005; Railo *et al*, 2008). Wnt signalling through other FZDs might protect our transfectants from apoptosis.

Western blot analyses for MAP kinases revealed that the phosphorylation level of JNK was decreased whereas expression and phosphorylation levels of other kinases were not (Figure 2C). As expected from this result and the fact that *c-jun* is a Wnt target gene (Mann *et al*, 1999), expression and phosphorylation of *c-Jun* were also reduced (Figure 2C). A recent finding that RhoA stimulates c-Jun expression through ROCK, which in turn activates JNK (Marinissen *et al*, 2004), prompted us to examine RhoA activation. As shown in Figure 2D, it was clearly decreased with FZD7_siRNA transfection, suggesting that FZD7 may be a receptor for the non-canonical Wnt/JNK signalling pathway in colon cancer cells. These data may provide a molecular explanation for the previous findings that inhibition of FZD7 expression decreased the migratory activity of colon cancer cells (Vincan *et al*, 2007) and hapatocellular carcinoma cells (Merle *et al*, 2004).

We have also demonstrated decreased invasion activity of FZD7-down-regulated HCT-116 cells (Figure 2E). In addition to migratory activity, proteolytic lysis of Matrigel is required for cells to penetrate the membrane in the invasion assay we used. The canonical Wnt pathway may be responsible for this process, because it involves several extracellular proteinases such as urokinase-type plasminogen activator (uPA) (Hiendlmeyer *et al*, 2004), uPA receptor (Mann *et al*, 1999), CD44 (Wielenga *et al*, 1999), matrix metalloproteinase (MMP)-7 (Brabletz *et al*, 1999) and MT1-MMP/MMP-14 (Takahashi *et al*, 2002). It is also known that the *β*-catenin/Tcf target gene *fra-1* directly induces MMP-1 and MMP-9 promoter activity (Belguise *et al*, 2005). Our quantitative RT-PCR data showed that the expression levels of CD44v8-9 and MT1-MMP were decreased after FZD7_siRNA transfection into HCT-116 cells (Figure 2F).

It remains to be determined how non-canonical Wnt signalling interacts with the canonical Wnt/*β*-catenin/Tcf signalling in colon cancer cells. It was shown that inhibitors of the canonical signalling, Dickkopf (DKK)-1 and a dominant-negative Tcf construct, did not reduce Wnt3a-dependent motility of CHO-K1 cells (Endo *et al*, 2005), and that DKK-1 and DKK-2 had no effect on Wnt3a-induced migration of myeloma cells (Weeraratna *et al*, 2002). In the former cell model system, RhoA was also activated with Wnt3a, whereas the activation of PKC family proteins including PKC*α*, PKC*β* and PKC*μ* as well as RhoA was found in the latter. These findings suggest that cell migration stimulated with Wnt may be independent of canonical signalling.

We have shown that stable transfectants of HCT-116 cells harbouring the FZD7_siRNA have decreased Tcf activity, viability and invasion (Figures 3B–D), and less *in vivo* metastatic activity using a liver metastasis model of HCT-116 cells in nude mice (Bouvet *et al*, 2006). The number of metastatic foci with FZD7_siRNA transfectants was significantly decreased compared to that with control cells (Figure 3E). A major molecular basis for this suppressive effect is thought to be due to the decreased expression of motility- and invasion-related genes, but it is possible that it partly reflects the reduced cell viability. As described above, it was reported that FZD7 promotes cell survival without altering cell proliferation in hMSCs (Song *et al*, 2006). Furthermore, canonical Wnt signalling was shown to be involved in the regulation of proliferation, as well as the migration/invasion capacity of hMSCs (Neth *et al*, 2006). In this context, FZD7 might be one of mesenchymal characteristics of colon cancer cells when they metastasise through epithelial–mesenchymal transition (Turley *et al*, 2008).

An important finding of this report is the prognostic significance of FZD7 mRNA expression in primary CRC tissues. FZD7 expression was higher in the Recurrence+Death group than in the Disease-free group (Figure 4D), and patients with higher FZD7 expression levels (⩾mean of all cases) had worse overall survival (Figure 4C). However, no association was found between FZD7 expression and clinico-pathological factors except for pathological stage. This may be related to the functional diversity of FZD7 including induction of mesenchymal–epithelial transition (Vincan *et al*, 2007), osteogenic differentiation of hMSCs (Song *et al*, 2006) and Wnt11 induction of differentiated cell phenotypes (Yamanaka and Nishida, 2007; Kim *et al*, 2008). We have detected Wnt11 mRNA in seven colon cancer cell lines (data not shown). Nevertheless, our present clinical data support the importance of FZD7 as a therapeutic target for CRC in those patients with higher FZD7 expression.

In conclusion, we first reported that FZD7 may be important in the survival, invasion and metastatic capabilities of colon cancer cells, at least partly, through expression of c-Jun, phosphorylation of c-Jun and JNK, and activation of RhoA. Furthermore, higher expression levels of FZD7 mRNA in primary CRC tissues were shown to be associated with poor prognosis, suggesting that FZD7 may be involved in CRC progression.

## Figures and Tables

**Table 1:** Characteristics of colorectal cancer patients

	**All patients (*n*=135)**	**Selected patients**^a^ **(*n*=121)**
Age (years) (mean±s.d.)	66.9±11	66.4±11
		
*Sex*		
Male	70 (52%)	64 (53%)
Female	65 (48%)	57 (47%)
		
*pStage*		
I	14 (10%)	13 (11%)
II	51 (38%)	43 (35%)
III	42 (31%)	40 (33%)
IV	28 (21%)	25 (21%)
		
*pT*		
is	1 (1%)	1 (1%)
1	7 (5%)	7 (6%)
2	9 (7%)	8 (6%)
3	105 (78%)	93 (77%)
4	13 (9%)	12 (10%)
		
*pN*		
(−)	74 (55%)	63 (52%)
(+)	61 (45%)	58 (48%)
		
		
*pM*		
(−)	108 (80%)	95 (79%)
(+)	27 (20%)	26 (21%)
		
*Pathology*		
Moderately differentiated adenocarcinoma	81 (60%)	72 (60%)
Well-differentiated adenocarcinoma	42 (31%)	39 (32%)
Poorly differentiated adenocarcinoma	7 (5%)	6 (5%)
Mucinous adenocarcinoma	5 (4%)	4 (3%)

Abbreviations: pT=pathological tumor classification; pN=lymph node invasion; pM=distant metastasis.

a The patients whose follow-up data were available.
